# Analysing the essential proteins set of *Plasmodium falciparum* PF3D7 for novel drug targets identification against malaria

**DOI:** 10.1186/s12936-021-03865-1

**Published:** 2021-08-03

**Authors:** Fawad Ali, Hira Wali, Saadia Jan, Asad Zia, Muneeba Aslam, Imtiaz Ahmad, Sahib Gul Afridi, Sulaiman Shams, Asifullah Khan

**Affiliations:** 1 Department of Biochemistry, Abdul Wali Khan University Mardan, Mardan, 23200 Pakistan; 2 Department of Biochemistry, Hazara University, Mansehra, 21120 Pakistan

**Keywords:** *Plasmodium falciparum*, Malaria, Comparative proteomics, Anti-malarial therapeutics, Pharmacophore modelling

## Abstract

**Background:**

*Plasmodium falciparum* is an obligate intracellular parasite of humans that causes malaria. Falciparum malaria is a major public health threat to human life responsible for high mortality. Currently, the risk of multi-drug resistance of *P. falciparum* is rapidly increasing. There is a need to address new anti-malarial therapeutics strategies to combat the drug-resistance threat.

**Methods:**

The *P. falciparum* essential proteins were retrieved from the recently published studies*.* These proteins were initially scanned against human host and its gut microbiome proteome sets by comparative proteomics analyses. The human host non-homologs essential proteins of *P. falciparum* were additionally analysed for druggability potential via in silico methods to possibly identify novel therapeutic targets. Finally, the PfAp4AH target was prioritized for pharmacophore modelling based virtual screening and molecular docking analyses to identify potent inhibitors from drug-like compounds databases.

**Results:**

The analyses identified six *P. falciparum* essential and human host non-homolog proteins that follow the key druggability features. These druggable targets have not been catalogued so far in the Drugbank repository. These prioritized proteins seem novel and promising drug targets against *P. falciparum* due to their key protein–protein interactions features in pathogen-specific biological pathways and to hold appropriate drug-like molecule binding pockets. The pharmacophore features based virtual screening of Pharmit resource predicted a lead compound i.e. MolPort-045–917-542 as a promising inhibitor of PfAp4AH among prioritized targets.

**Conclusion:**

The prioritized protein targets may worthy to test in malarial drug discovery programme to overcome the anti-malarial resistance issues. The *in-vitro* and *in-vivo* studies might be promising for additional validation of these prioritized lists of drug targets against malaria.

**Supplementary Information:**

The online version contains supplementary material available at 10.1186/s12936-021-03865-1.

## Background

Malaria is a life-threatening infectious disease caused by parasitic protozoan plasmodium. It is a vector borne disease, transmitted to humans through the bite of infected carrier female *Anopheles* mosquitoes. Among five parasite species that cause malaria in humans, two species, *Plasmodium falciparum* and *Plasmodium vivax,* have the greatest threat to human life [1]. The *P. falciparum*, a unicellular protozoan, belongs to the family Plasmodiidae and lies in the phylum Apicomplexa [2]. *Plasmodium falciparum* alone is responsible for almost all malaria-inflicted deaths in sub-Saharan Africa, with the continent bearing over 90% of the global *P. falciparum* burden [3–5]. Asia is second to Africa in terms of malaria prevalence. In 2019, the World Health Organization (WHO) estimated 229 million malaria cases and about 409,000 deaths due to malaria worldwide every year [6]. More than 85% of confirmed recorded cases and deaths in Asia occurred in India, Indonesia, Myanmar, and Pakistan [7].

The *P. falciparum* resistance is reported to many approved anti-malarial drugs, including the chloroquine and artemisinin [8]. Resistance to chloroquine was first observed in Thailand in 1957 and the Colombian-Venezuelan border in 1959 [9]. Drug resistance had spread across sub-Saharan Africa by 1988, and today chloroquine is no longer effective in almost all parts of the world [10]. Specific polymorphisms in the *P. falciparum* chloroquine resistance transporter (PfCRT) are reported in association with chloroquine resistance [11]. Likewise, the artemisinin resistance was first documented in 2008 in the Thailand-Cambodia border regions [12, 13]. The artemisinin resistance associated with delayed parasitic clearance after three days of artemisinin monotherapy. Several studies have reported that artemisinin resistance emerged due to a polymorphism in the *pfk13* gene [14, 15].

The indispensable proteins of *P. falciparum* recently explored from well experimental approaches in some studies. These repositories are promising to identify suitable targets to overcome the drug-resistant *P. falciparum* infection [16, 17]. Zhang et al*.* [16] experimentally analysed 5399 genes and identified 2680 as essential for optimal growth of *P. falciparum* during asexual blood stages. These essential genes coding the *P. falciparum* vital proteins, including drug targets and potential vaccine candidates. Besides, there are over 1,000 of *Plasmodium*-conserved essential genes with unknown biological functions so far. In the current study, these essential proteins were retrieved from the two recently published studies and assessed for druggable potential based on comparative proteomics, protein–protein interactions and drug-like molecules binding potential.

## Methods

**Fig. 1:**
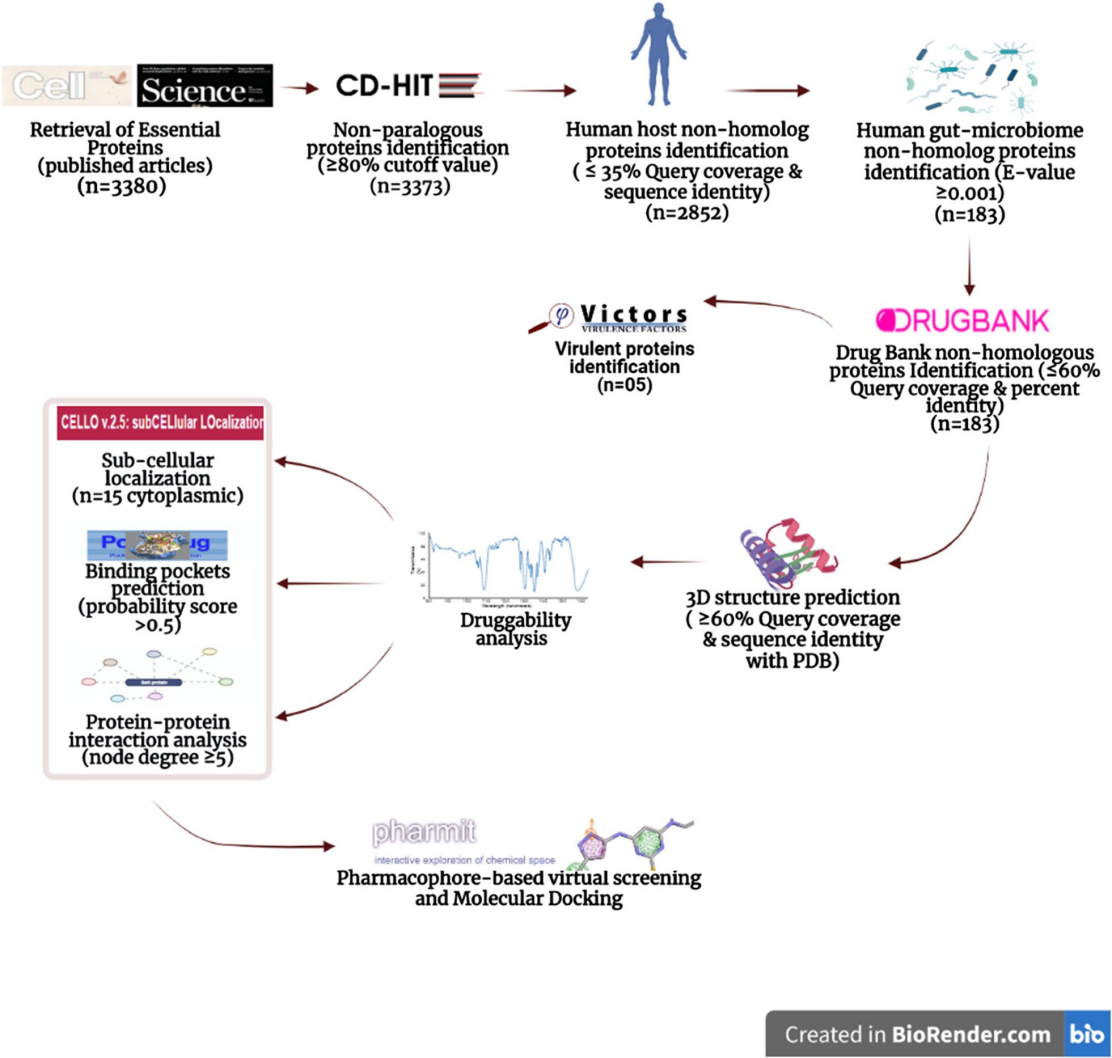
The stepwise workflow adopted for novel anti-malarial drug targets identification

The methodological layout of the current study is depicted in Fig. 1.

### Retrieval of non-paralogous essential proteins

The essential proteins of *P. falciparum* strain 3D7 were retrieved from two recently published studies [16, 17]. The paralogous proteins were removed by CD-HIT clustering analysis with  ≥ 80% sequence similarity cutoff [18].

### Human host non-homologous and virulent proteins identification

The *P. falciparum* essential proteins non-homolog to human host proteome were identified by comparative sequence analyses via BLASTp tool [19]. The threshold values of  ≤ 35% query coverage and sequence identity were set during this analysis [20]. The proteins having significant similarity with human proteome were discarded and the remaining non-homologs were shortlisted for further analysis. The non-homology search against human gut microbiota proteins sequences was also carried out with a threshold cutoff, i.e. E value ≥ 0.001 [21, 22]. The Victors database was screened for *P. falciparum* 3D7 virulent proteins annotation. The Victors database contains 5304 virulent proteins data from various parasites including *P. falciparum* [23].

### Drugbank database scanning

The shortlisted essential proteins of *P. falciparum* from above analyses were scanned against the Drugbank database to identify novel drug targets with ≤ 60% query coverage and percent identity threshold of BLASTp [24].

### Structure homologs search

The proteins data bank (PDB) was screened to identify the homologous 3D structures of pathogenic proteins [25]. The pathogen proteins were BLAST against the entire PDB database entries [22, 26]. The pathogen sequences having  ≥ 60% homology were modeled with Swiss Model [27] and verified by ERRAT [28] and RAMPAGE [29].

### Druggablity analyses

The prioritized list of essential proteins shortlisted from above analyses were tested for druggability potential. The drug-like molecules binding pockets of the targets were identified by PockDrug-server [30]. The subcellular localization was performed with CELLO v.2.5 [31]. The molecular weight of the proteins were also accounted during their druggability assessment [32]. The PPI analysis was performed by the STRING database and the Hub proteins were identified based on node degree (K ≥ 5) that represents the number of interactions [33].

### Pharmacophore based virtual screening

Pharmit server was employed to design a pharmacophore model using the 3D structure of *P. falciparum* diadenosine tetraphosphate hydrolase (PfAp4AH) enzyme (PDB ID 5CFJ) [43]. Pharmit provides interactive screening of millions of chemical compounds from built-in databases, i.e. Molprot, ChEMBL, ZINC, and PubChem. The Pharmit server is based on a pharmacophore model using AutoDock Vina scoring function [34]. The pharmacophore model was built using seven features, i.e. two hydrogen donors, two hydrogen acceptors, two hydrophobic features, and one aromatic feature. The screening results were then minimized to a significant level based on Pharmit scoring and RMSD values to obtain the best possible inhibitors out of millions of drug-like compounds. The top ten hits identified based on Pharmit screening were then docked using the CB-Dock tool to verify the ligand-binding sites [35]. Discovery studio was used for protein–ligand interaction visualization [36].

## Results

### Subtractive proteomic analyses

Essential genes perform key cellular functions for the survival of pathogens [37]. The *P. falciparum* strain 3D7 essential genes information were obtained from the recently published articles [16, 17] and total 3380 essential proteins were identified. These proteins sequences were retrieved from Uniprot. Seven paralogous protein sequences were excluded by CD-HIT analysis and the remaining 3373 sequences were considered for downstream analysis (Additional file 1: Table S1). The non-paralogous protein sequences were subjected to BLASTp against human proteome as well as human gut microbiome proteome data with threshold parameters. The gut flora is helpful to the host in many ways like vitamins biosynthesis and absorption of short chain-fatty acids [38]. The unintended inhibition of gut microbe leads to a decrease in gut flora and colonization of pathogenic bacteria in the host gut [39]. The comparative sequence analysis based screening (see, methodology section) against human proteome identified total 2852 *P. falciparum* proteins non-homolog to human host. Further screening of these *P. falciparum* essential and human host non-homolog proteins against human gut microbiome proteins database identified 2669 homologs and 183 non-homologs proteins (Fig. 1; Additional file 2: Table S2; Additional file 3: Table S3). The five among these 183 were annotated as *P. falciparum* 3D7 virulent proteins during screening of Victors database (Table 1). The 183 *P. falciparum* essential proteins identified as human as well as human gut proteome non-homologs were prioritized for downstream analyses. The homology screening of these 183 proteins based on set threshold against Drugbank repository inferred no homology with already reported drug targets deposited in drugbank database. However, 38 established anti-malarial targets from Alexander et al. [77] (Additional file 4: Table S4) were additionally catalogued to check the recovery of previously reported targets in current study. Among approved anti-malarial targets, the *P. falciparum* 2-C-methyl-D-erythritol 4-phosphate cytidylyltransferase and *P. falciparum* subtilisin-like protease-1 were listed within the 183 prioritized targets (Additional file 2: Table S2). However, when the cutoff criteria was relaxed to 35% sequence identity and 35% query coverage to screen the human gut microbiome database, then additional 16 already approved anti-malarial targets are recovered (Additional file 4: Table S4). The recovery of these established anti-malarial targets somehow validates the strategy been acquired in current study.

**Table 1:** The *P. falciparum* 3D7 essential virulent proteins, non-homolog to human host as well as human gut microbiome proteome

	Protein accession number	Protein name	Nature of protein
1	Q9TY99_PLAF7	knob-associated histidine-rich protein	Virulent
2	Q8I5P1_PLAF7	Cell traversal protein for ookinetes and sporozoites (CelTOS), putative	Virulent
3	C6KTB1_PLAF7	4-methyl-5(B-hydroxyethyl)-thiazol monophosphate biosynthesis enzyme	Virulent
4	Q8I6Z5_PLAF7	Plasmepsin V	Virulent
5	Q8IDM6_PLAF7	Nucleoside transporter 1	Virulent

### Druggability analyses

**Fig. 2:**
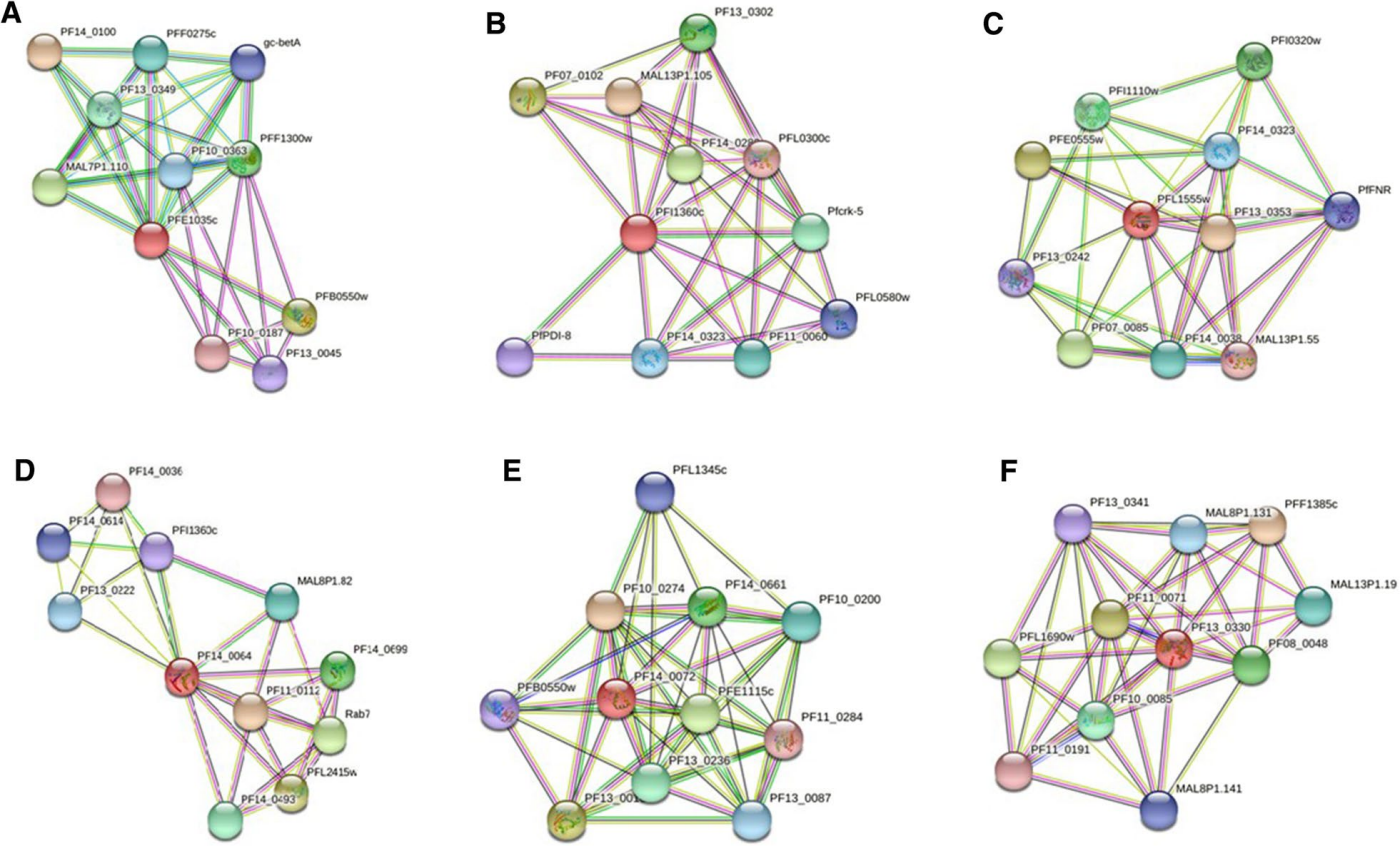
Protein–protein interaction plots of best interacting proteins. The red colour indicates query protein. **A** Bis(5'-nucleosyl)-tetraphosphatase. **B** Serine/threonine-protein phosphatase. **C** Cytochrome b5, putative. **D** Vacuolar protein sorting-associated protein 29. **E** Multifunctional methyltransferase subunit TRM112, putative. **F** RuvB-like helicase. All these proteins showed vital interaction with other *P. falciparum* proteins

The Drugbank non-homologous proteins were prioritized for downstream druggability analyses. The subcellular localization is one of the key aspect of druggability and the cytoplasmic proteins are considered as suitable drug targets [40, 41]. The 15 *P. falciparum* proteins among shortlisted prioritized targets were annotated as cytoplasmic proteins. The proteins 3D structures identified by Swiss model were validated with the ERRAT tool with quality factor score of > 50, which is accepted as high quality model [42]. Ramachandran plot identified 80–90% of modelled proteins residues in the allowed region assuring good quality structure modelling of the target proteins (Table 2). Finally, six (06) proteins were prioritized on the basis of (i) pockdrug probability score ˃0.5 [30], (ii) ERRAT quality factor ≥ 90 [28], and (iii) protein–protein interaction node degree i.e. K ≥ 5 [33] (Table 2; Fig. 2). These six prioritized targets are speculating to hold promising druggable pockets to anchor small drug-like molecules and act as indispensable hub proteins in *P. falciparum* metabolic network.

**Table 2:** Druggability analyses of shortlisted 15 cytoplasmic proteins of *P. falciparum* 3D7 essential and human host non-homolog proteins

Uniprot accession numbers	PDB homolog ID’s	Protein names	ERRAT quality factor	Ramachandran plot (residues in favored region %)	QMEAN > -4	Pock drug score > 0.5 (Residues in pocket)	Molecular weight (Dalton)	Node degree (K) ≥ 5 (STRING analysis)
O97259_PLAF7	3fga.1.C	Serine/threonine-protein phosphatase	99.3103	94.75%	− 2.11	0.24 (15)	35,765.00	7.45
C0H4F3_PLAF7*	5cfi.1.A	Bis(5'-nucleosyl)-tetraphosphatase [asymmetrical]	96.1832	97.20%	- 0.37	0.98 (8)	17,750.19	6.36
Q8I3A9_PLAF7	4nc6.1.A	GTPase-activating protein, putative	90.2778	90.71%	− 3.37	0.99 (20)	50,301.05	4.36
Q8I2N2_PLAF7*	4lac.1.C	Serine/threonine-protein phosphatase	93.5484	97.54%	− 0.72	0.91 (14)	36,386.98	6.18
Q8I082_PLAF7	1ryt.1.A	Rifin	69.7368	93.41%	− 2.95	0.32 (10)	40,648.33	3.82
Q8IIU3_PLAF7	2c9o.1.A	RuvB-like helicase	88.7505	92.14%	− 2.41		53,405.94	7.45
Q8I599_PLAF7*	2ibj.1.A	Cytochrome b5, putative	94.5205	95.00%	− 0.14	1 (19)	18,244.08	6.55
Q8IDG3_PLAF7	6muw.1.C	Proteasome subunit alpha type	92.2727	93.72%	− 1.63		27,947.73	10
Q8IM27_PLAF7*	2a22.2.A	Vacuolar protein sorting-associated protein 29	92.3977	95.63%	0.23	0.96 (16)	21,774.02	5.09
Q8IM19_PLAF7*	6k0x.1.B	Multifunctional methyltransferase subunit TRM112, putative	92.233	91.80%	− 1.87	1 (10)	14,234.31	8.18
Q8ILZ5_PLAF7	6y4l.1.A	ER membrane protein complex subunit 2	100	95.92%	− 2.03	0.97 (16)	34,952.82	4.36
C6S3I2_PLAF7	6t69.1.A	MORN repeat-containing protein 5	68.3333	87.14%	− 0.84	0.98 (12)	17,938.95	1.33
Q8IKK5_PLAF7	1w98.1.B	Mediator of RNA polymerase II transcription subunit 20, putative	88.6179	93.80%	− 3.17	0.99 (11)	36,709.76	
Q8IKC5_PLAF7	4l02.1.A	Diacylglycerol kinase	85.8025	81.58%	− 8.09	0.98 (32)	57,642.42	7.09
Q8ID85_PLAF7*	4ww4.1.B	RuvB-like helicase	91.3242	94.46%	− 1.49	1 (14)	54,629.36	7.27

The steric* indicates shortlisted 06 best druggable targets

### Pharmacophore based virtual screening

**Fig. 3:**
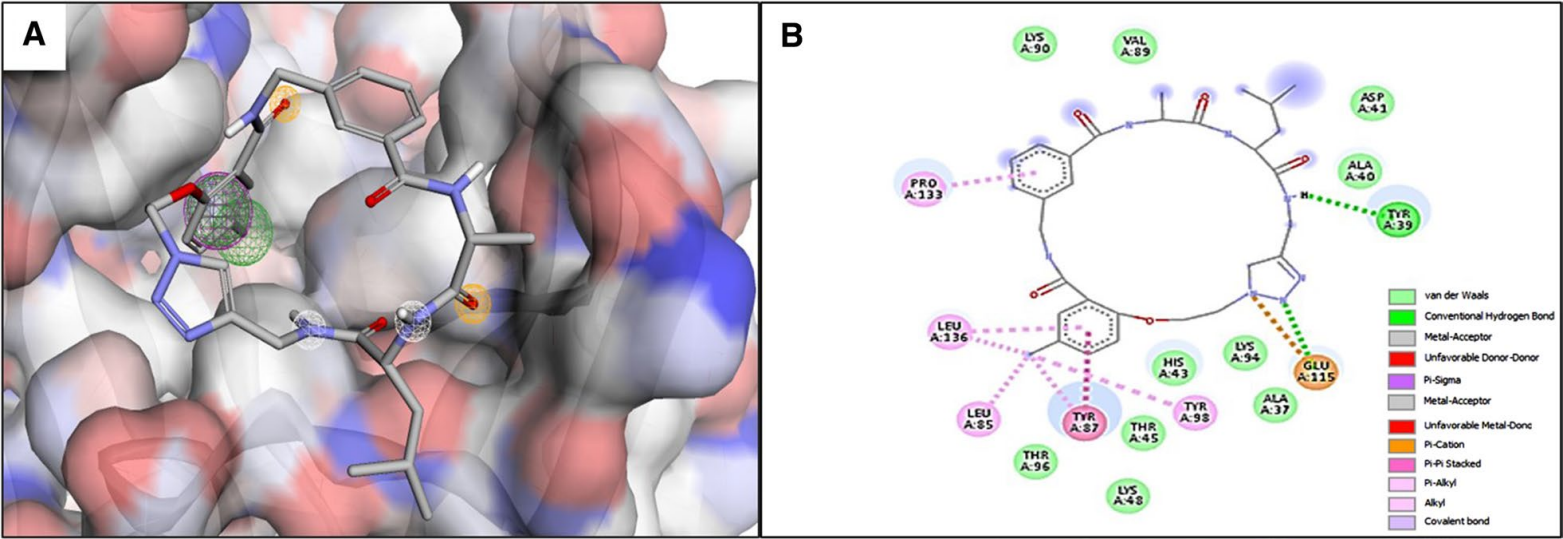
**A** Pharmacophore model designed based on the active site of the PfAp4AH enzyme. The features indicated with different colours. Hydrogen-bond donor (white), hydrogen acceptor (yellow), hydrophobic features (green) and aromatic (pink) **(B)** The molecular interactions of top compound docked within the substrate binding site of PfAp4AH. The nature of protein–ligand interactions shown with different colours legends

Among finally shortlisted targets, the PfAp4AH was prioritized for drug-like compounds virtual screening. The pharmacophore model designed from the three-dimensional structure of ligand binding pocket of PfAp4AH showed seven features, i.e. two hydrogen donors, two hydrogen acceptors, two hydrophobic features, and one aromatic feature shown in Fig. 3A. Top 10 hits acquired from Pharmit resource screening were prioritized based on score and RMSD values (Table 3). These top hits compounds were then docked within the ligand binding pocket of PfAp4AH enzyme to predict their binding conformation. The 3D structure of the PfAp4AH enzyme reported with bound substrate (i.e. ATP) within active-site. The site comprises of seven key residues, i.e. Tyr87, Lys94 Ser135, Pro133, His43, Lys48, and Glu115, responsible for ATP hydrolysis [43]. This active-site was utilized in the current study for pharmacophore-based virtual screening. The three key residues of this active-site, i.e. Tyr87, Glu115, and Pro133 are found to primarily engage with the binding of top hit compound (i.e. MolPort-045–917-542) (Fig. 3B). The conserved Glu115 residue is reported as especially important for hydrolysis reaction of PfAp4AH [43]. Besides, the docking analyses predicted that all the top screened compounds explicitly anchor to this active site.

**Table 3:** The top 10 best docked compounds of Pharmit resource ranked based on the CB-dock score, Pharmit score and RMSD values

Docked ligand	Pharmit IDs	Chemical structure	CB-Dock score	Pharmit score	RMSD value
1	MolPort-045–917-542		− 10.6	− 8.53	2.8
2	MolPort-045–918-058		− 8.1	− 8.02	2.6
3	MolPort-045–931-878		− 8.4	− 6.98	2.5
4	MolPort-045–915-346		− 9.2	− 6.90	2.9
5	MolPort-045–934-296		− 8.4	− 6.75	2.9
6	MolPort-035–379-921		− 8.2	− 6.66	2.7
7	MolPort-046–894-718		− 7.5	− 6.66	2.6
8	MolPort-045–918-093		− 9.3	− 6.65	2.9
9	MolPort-019–706-160		− 7.5	− 6.64	2.0
10	MolPort-029–999-758		− 8.5	− 6.61	2.8

## Discussion

In the current study, the 3380 essentially reported proteins of *P. falciparum* strain 3D7 were analysed to address potent novel druggable targets. These proteins were analysed based on their non-homology with the human host as well as human gut microbiome proteome. The targets were additionally shortlisted based on strict threshold criteria of basic druggability features. Among the shortlisted targets (Table 2), the protein, Bis(5'-nucleosyl)-tetraphosphatase (asymmetrical) (C0H4F3_PLAF7) also known as asymmetrical diadenosine 5′,5″-P1,P4-tetraphosphate hydrolase (PfAp4A) (EC 3.6.1.17) enzyme participates in pyrimidine and purine metabolism [44, 45]. The PfAp4A hydrolase exhibited high-temperature stability even at 60 °C [46]. Previously in few studies, the PfAp4A is also tested as a potential drug target against *P. falciparum* [47, 48].

The protein serine/threonine-protein phosphatase (Q8I2N2_PLAF7) was also found among finally shortlisted target that involve in regulation of many cellular signaling pathways by catalysing the removal of phosphate group from target enzymes. This enzyme plays a central role in the functional regulation and control of different genes related to the cell cycle [49]. The phosphorylation regulates several primary steps in *P. falciparum's* diverse life cycles. Many of the kinases and phosphatases as well as their substrates are specific to parasites, making eventually the phosphorylation event as a viable target for anti-parasitic action [50]. The protein phosphatase-1, a type of PfPP, involve in the mitotic division of *P. falciparum* and plays an important role in the liberation of merozoites. Prior studies on *P. falciparum* revealed that the activity of PfPP1 is more important as compared to protein phosphatase 2A (PP2A) [51]. This also verified by transcriptomic analysis, where the PfPP1 transcript levels reported higher than PP2A after 24 h of RBC infection [52]. The okadaic acid (OA), a toxin initially isolated from a marine sponge, i.e. *Halichondria okadai* has been identified as a selective inhibitor of serine/threonine protein phosphatases (PPPs) and reported to strongly inhibits the PP1, 2A, and 2B in-vitro [53]. Out of 30 examined protein phosphatase, the 16 protein phosphatases along with PP1 and putative phosphatases seem to be important for blood-stage parasites [51]. Moreover, some studies also showed that PfPP1 is indispensable for blood-stage parasite survival [54]. Many phosphatases play key roles in the pathological pathways, and their inactivation may help to prevent or postpone the emergence of human diseases. Therefore, the potent inhibitors for such phosphatases might be of great therapeutic benefit.

The enzyme cytochrome b5 Reductase (cb5r) (Q8I599_PLAF7) plays a role in fatty acid elongation, cholesterol biosynthesis, and cytochrome P450-mediated detoxification of xenobiotics [55]. This protein has been thoroughly studied in mammals, but still needs to be characterized in microorganisms, such as fungi and parasites, including *P. falciparum*. There is a close phylogenetic relationship between the plant and *P. falciparum* cb5r proteins. The plant cb5r has been identified as a novel herbicidal target [55]. This protein reported essential for *P. falciparum* survival and was found human host non-homolog and possibly be a potent therapeutic target, thereby might be a worthy candidate for drug development against malaria.

The vacuolar protein sorting-associated protein 29 (VPS29) **(**Q8IM27_PLAF7**)** is involved in the essential metabolic process of proteins translocation to the subcellular organelles. The *P. falciparum* sort and traffic newly synthesized proteins to target intracellular organelles as well as beyond the plasma membrane into the host cell in some cases [56]. The *P. falciparum* VPS29 (i.e. PfVPS29) is the functional component in the assembly of the retromer complex [57]. During the PPI analysis, the pfVPS29 showed direct interactions with other retromer complex components i.e. PfVPS26, VPS9, VPS10 as shown in Fig. 2. The PfVPS29 is located in the cytosol and highly expressed in early trophozoite and schizont stages [58]. Inhibiting the activity of PfVPS29 may lead to the disassembling of the retromer complex and possibly halt the protein sorting function of the *P. falciparum*.

The multifunctional methyltransferase subunit **(**Q8IM19_PLAF7**)** have methyltransferase activity during post-translational modifications, chromatin remodeling and protein heterodimerization activity [44]. The protein methyltransferases (PMTs) have been linked to the pathogenesis of a variety of diseases, including human cancers, inflammatory diseases, metabolic diseases, and neurodegenerative diseases. The PMTs are highly attractive among the histone-modifying enzymes and act as drug targets [59, 60]. However, to date no study been conducted about the inhibition of *P. falciparum* methyltransferase.

The RuvB-like helicase (Q8ID85_PLAF7) also shortlisted as therapeutic target in the current study. The RuvB-like helicase function like ATP- dependent helicases. It has a vital role in the cell cycle and transcription [61–63]. The RUVBL proteins (RUVBL1 & 2) are known to regulate various essential cellular processes in different organisms like *Saccharomyces cerevisiae*, *Drosophila melanogaster* and *Caenorhabditis elegans* [64–66]. Three types of RuvB, i.e., PfRuvB1, PfRuvB2, and PfRuvB3 are present in the *P. falciparum*. The PfRuvB1 possesses ssDNA-stimulated ATPase activity and function as a helicase that unwind the DNA in a 5' to 3' direction [62]. The PfRuvB2 function similar to PfRuvB1, however, its helicase activity is comparatively weak. The PfRuvB3 function only as ATPase with no helicase activity during schizont/merozoits or interaerythocytic mitosis [67]. During the developmental stages of the parasite, the PfRuvB1 and PfRuvB2 are expressed in the asexual phase, while the PfRuvB3 expresses only during the schizont stage, where intraerythrocytic mitosis of *P. falciparum* occurs [68]. The PfRvuBL3 protein is a true homolog of yeast RuvBL2. Since in yeast, the RuvBL proteins are found extremely indispensable for survival and known to regulate the transcription of almost 5% of yeast genes [64]. The RuvB- like helicases are suitable drug targets to control malaria due to their essentiality for pathogen and non-homology with human host proteome. It is reported that helicases are required for the proliferation of bacteria, viruses and *Plasmodium*, and inhibiting the DNA unwinding activity reduces the replication of these pathogens in cell cultures and animal models [69–71]. The PfRuvB1 ATPase activity is formerly reported to be inhibited by actinomycin, novobiocin, and ethidium bromide [72].

Among the shortlisted targets, the refined 3D structure of PfAp4AH is available in PDB. It was, therefore, prioritized for drug-like compounds screening based on in silico drug discovery approaches to address potent inhibitors. The PfAp4AH enzyme regulates the levels of signaling molecules, i.e. diadenosine tetraphosphate (Ap4A) by hydrolyzing it to ATP and AMP. This enzyme is localized at the infected RBC membrane in the subpopulation of infected cells [43]. The Ap4A and Ap5A molecules are the chief substrates of PfAp4AH enzyme and key mediators of cellular communication and function through purinergic receptors [73]. Hence, signaling mediated by these molecules within RBCs is of special interest in malaria [74]. The purinergic signaling event is reported to play an important role in parasite invasion [75]. Limited or no comprehensive studies are available about the PfAp4AH inhibition and drug-like compound screening [74]. However, this target been considered worthy for drug discovery and inhibition in other species, such as *Mycobacterium tuberculosis* [76].

## Conclusion

The recently published essential proteins of *P. falciparum* were utilized and the comparative proteomics analyses along with in silico druggability approaches were employed to identify novel and suitable drug targets against *P. falciparum*. The study based on comparative sequence analysis, updated biological databases scanning and multi-direction druggability analyses. This ultimately prioritized and addressed several novel druggable targets against *P. falciparum* infection not highlighted before. The drug targets and the drug-like compounds prioritized in the current study would be worthy to devise new strategies to combat the *P. falciparum* drug resistance issues.

## Supplementary Information

**Additional file 1: ***Plasmodium falciparum* strain 3D7 non paralogous essential proteins.

**Additional file 2 : ***Plasmodium falciparum* strain 3D7 proteins, non-homologous to human as well as human’s gut flora proteins.

**Additional file 3 :*** Plasmodium falciparum* 3D7 essential proteins homologs to human gut microbiome proteome.

**Additional file 4: **The approved antimalarial targets obtained from Alexander, S. P., et al. (2019) [77]. The accession IDs in Bold show approved antimalarial targets recovered in the list of 183 prioritized targets (Table S2), while accession IDs in italic and asterisk* are approved targets recovered by relaxing the human microbiome database screening criteria to 35% sequence identity and query coverage.

## Data Availability

The supplementary data relevant to this study is provided.

## Abbreviations

WHO: World Health Organization
PfCRT: *Plasmodium falciparum* Chloroquine resistant transporter
Pfk13 gene: *Plasmodium falciparum* Kelch-13 gene
BLAST: Basic Local Alignment Search Tool
Ap4AH: Diadenosine tetraphosphate Hydrolase
PDB: Protein Data Bank
PfPP: *Plasmodium falciparum* Protein phosphatase
