# Supplementary material for: Analysing the essential proteins set of Plasmodium falciparum PF3D7 for novel drug targets identification against malaria
Source: Malar J. 2021 Aug 3;20:335. doi: 10.1186/s12936-021-03865-1 (PMC8336052; doi:10.1186/s12936-021-03865-1)
Supplement: Supplementary file 4 — Additional file 4: The approved antimalarial targets obtained from Alexander, S. P., et al. (2019) [77]. The accession IDs in Bold show approved antimalarial targets recovered in the list of 183 prioritized targets (Table S2), while accession IDs in italic and asterisk* are approved targets recovered by relaxing the human microbiome database screening criteria to 35% sequence identity and query coverage. [file 12936_2021_3865_MOESM4_ESM.docx]

**Supplementary Table S4:**

The approved antimalarial targets obtained from *Alexander, S. P., et al.* (2019) [77]. The accession IDs in Bold letters show approved antimalarial targets present in the list of 183 prioritized targets (Supplementary Table S2), while accession IDs in italic and asterisk* are the 16 approved targets recovered by relaxing the human microbiome database screening criteria cutoff parameters to 35% sequence identity and 35% query coverage.

| S. No | Target ID | UniprotKB Accession code | Gene code | Enzyme Name |
| --- | --- | --- | --- | --- |
| 1 | 3168 | Q8I5G6 (Pf3D7) | api-IRS (Pf3D7) | Plasmodium falciparum isoleucine--tRNA ligase |
| 2 | 3059 | Q8IDJ8 (Pf3D7) | KRS1 (Pf3D7) | Plasmodium falciparum lysine--tRNA ligase |
| 3 | 2954 | Q8I246 (Pf3D7) | cPheRS (Pf3D7) | Plasmodium falciparum phenylalanine--tRNA ligase alpha subunit |
| 4 | 3056 | Q8I5R7 (Pf3D7) | PRS (Pf3D7) | Plasmodium falciparum proline--tRNA ligase |
| 5 | 3058 | Q8IIA4 (Pf3D7) | ThrRS (Pf3D7) | Plasmodium falciparum threonine--tRNA ligase |
| 6 | 3166 | A0A144A140 (Pf3D7) | aTrpRS (Pf3D7) | Plasmodium falciparum tryptophan--tRNA ligase |
| *7* | 3089 | *Q8ILP4 (Pf3D7)** | PANK1 (Pf3D7) | Plasmodium falciparum pantothenate kinase 1 |
| 8 | 2981 | Q8I1R6 (Pf3D7) | DHFR-TS (Pf3D7) | Plasmodium falciparum bifunctional dihydrofolate reductase-thymidylate synthase |
| *9* | 3065 | *Q8IAU3 (Pf3D7)** | PPPK-DHPS (Pf3D7) | Plasmodium falciparum hydroxymethyldihydropterin pyrophosphokinase-dihydropteroate synthase |
| 10 | 3066 | Q8I566 (Pf3D7) | SHMT (Pf3D7) | Plasmodium falciparum serine hydroxymethyltransferase |
| 11 | 3079 | Q8IL04 (Pf3D7) | HDP (Pf3D7) | Plasmodium falciparum heme detoxification protein |
| *12* | 3072 | *Q7K6A1 (Pf3D7)** | HDAC1 (Pf3D7) | Plasmodium falciparum histone deacetylase 1 |
| 13 | 3105 | Q8IIL5 (Pf3D7) | CLK3 (Pf3D7) | Plasmodium falciparum cyclin-dependent-like kinase CLK3 |
| 14 | 3013 | Q8I719 (Pf3D7) | PKG (Pf3D7) | Plasmodium falciparum cGMP-dependent protein kinase |
| *15* | 3073 | *Q8I3V5 (Pf3D7)** | PI3K (Pf3D7) | Plasmodium falciparum phosphatidylinositol 3-kinase |
| *16* | 2972 | *Q8I406 (Pf3D7)** | PI4KB (Pf3D7) | Plasmodium falciparum phosphatidylinositol 4-kinase |
| 17 | 3087 | Q02768 (Pf) | CYTB (Pf3D7) | Plasmodium falciparum cytochrome b |
| 18 | 3090 | Q8I302 (Pf3D7) | NDH2 (Pf3D7) | Plasmodium falciparum type II NADH:ubiquinone oxidoreductase |
| 19 | 2980 | Q8IKG4 (Pf3D7) | DXR (Pf3D7) | Plasmodium falciparum 1-deoxy-D-xylulose 5-phosphate reductoisomerase |
| 20 | 3064 | **Q8I273 (Pf3D7)** | IspD (Pf3D7) | Plasmodium falciparum 2-C-methyl-D-erythritol 4-phosphate cytidylyltransferase |
| 21 | 2949 | Q08210 (Pf3D7) | DHODH (Pf3D7) | Plasmodium falciparum dihydroorotate dehydrogenase |
| 22 | 3091 | Q8I5V4 (Pf3D7) | PDEalpha (Pf3D7) | Plasmodium falciparum cGMP-specific phosphodiesterase |
| 23 | 3077 | Q8I3X4 (Pf3D7) | PNP (Pf3D7) | Plasmodium falciparum purine nucleoside phosphorylase |
| *24* | 3174 | *C6KTB4 (Pf3D7)** | ACS (Pf3D7) | Plasmodium falciparum acetyl-CoA synthetase |
| *25* | 3173 | *Q8II79 (Pf3D7)** | FPPS/GGPPS (Pf3D7) | Plasmodium falciparum bifunctional farnesyl/geranylgeranyl diphosphate synthase |
| *26* | 3076 | *Q8IL83 (Pf3D7)** | CPSF3 (Pf3D7) | Plasmodium falciparum cleavage and polyadenylation specificity factor subunit 3 |
| *27* | 3075 | *C6KSZ4 (Pf3D7)** | GWT1 (Pf3D7) | Plasmodium falciparum GPI-anchored wall transfer protein 1 |
| 28 | 2955 | Q8ILW6 (Pf3D7) | NMT (Pf3D7) | Plasmodium falciparum glycylpeptide N-tetradecanoyltransferase |
| *29* | 3106 | *Q8I6Z5 (Pf3D7)** | PMV (Pf3D7) | Plasmodium falciparum plasmepsin V |
| 30 | 3071 | Q8IAS0 | PMX (Pf3D7) | Plasmodium falciparum plasmepsin X |
| *31* | 3088 | *Q8IJT1 (Pf3D7)** | PF3D7_1011400 (Pf3D7) | Plasmodium falciparum proteasome subunit beta type-5 |
| 32 | 3184 | **Q8I0V0 (Pf3D7)** | SUB1 (Pf3D7) | Plasmodium falciparum subtilisin-like protease 1 |
| *33* | 3074 | *C0H483 (Pf3D7)** | CARL (Pf3D7) | Plasmodium falciparum cyclic amine resistance locus protein |
| *34* | 2975 | *Q8IKW5 (Pf3D7)** | eEF2 (Pf3D7) | Plasmodium falciparum elongation factor 2 |
| *35* | 2971 | *A0A143ZZK9 (Pf3D7)** | ATP4 (Pf3D7) | Plasmodium falciparum non-SERCA-type Ca2+ -transporting P-ATPase |
| *36* | 3078 | *Q8IDS0 (Pf3D7)** | PF3D7_1341900 (Pf3D7) | Plasmodium falciparum V-type proton ATPase subunit D |
| 37 | 3069 | Q7KWJ5 (Pf3D7) | HT1 (Pf3D7) | Plasmodium falciparum hexose transporter |
| *38* | 3107 | *Q8I266 (Pf3D7)** | NCR1 (Pf3D7) | Plasmodium falciparum Niemann-Pick type C1-related protein |
